# Outcome of Involuntary Mental Health Assessment in a Psychiatric Department in Greece

**DOI:** 10.3390/healthcare11222977

**Published:** 2023-11-17

**Authors:** Vasiliki Papadopoulou, Aikaterini Arvaniti, Eleni Kalamara, Eugenie Georgaca, Stelios Stylianidis, Lily E. Peppou, Maria Samakouri

**Affiliations:** 1 Department of Psychiatry, Medical School, Democritus University of Thrace, 68100 Alexandroupolis, Greece; vasiliki.papadop3@gmail.com (V.P.); eleni.kalamara@euaa.europa.eu (E.K.); msamakou@med.duth.gr (M.S.); 2 University General Hospital of Alexandroupolis, 68100 Alexandroupolis, Greece; 3 European Asylum Support Office (EASO), 1917 Valletta MRS, Malta; 4 School of Psychology, Aristotle University of Thessaloniki, 54124 Thessaloniki, Greece; georgaca@psy.auth.gr; 5 Department of Psychology, Panteion University of Social Sciences, 17671 Athens, Greece; stylianidis.st@gmail.com (S.S.); lilly.peppou@gmail.com (L.E.P.)

**Keywords:** involuntary psychiatric admission, involuntary psychiatric hospitalization, coercion, predictive factors

## Abstract

Despite their controversiality, involuntary admissions in psychiatric departments remain a central issue in mental health care. The present study aims to identify demographic and clinical factors possibly associated with emergency involuntary psychiatric assessment and its outcome in Greece. This study was carried out in the psychiatric department of the University General Hospital of Alexandroupolis (UGHA) from 1 March 2018 to 28 February 2019. The sample included 191 individuals who had been psychiatrically assessed without their consent following a prosecutorial order. The majority of the involuntary assessments resulted in hospitalization (71%), with 51% of them resulting in involuntary hospitalization. Almost all patients diagnosed with “F20–29 schizophrenia, schizotypal and delusional disorders” were subsequently admitted to the psychiatric department of the UGHA (77 of 81, 66 of them involuntarily). Higher admission rates were recorded among those who had been referred from the Prosecutor’s Office of regions that are located far from the psychiatric department of UGHA (Fisher’s exact test, *p*-value = 0.045). In multivariate logistic regression, prior contact with psychiatric services and having an “F20–29 schizophrenia, schizotypal and delusional disorders” diagnosis was statistically significant with admission to the hospital as an outcome variable. Our study suggests an increased risk of involuntary admission among patients with psychosis, patients who had visited a psychiatric service prior to their assessment as well as those living further away from the main psychiatric services of the hospital. Better organization of community psychiatric services in remote places from hospital central services may lead to fewer prosecutorial referrals and coercive measures.

## 1. Introduction

Involuntary admission, i.e., the hospitalization of a patient with a mental disorder in a mental health unit without his or her consent, is a central issue in mental health care [1]. Ethical and legal debates have been raised worldwide, as coercive measures are applied and fundamental human rights are being affected: deprivation of liberty and disrespect of a person’s will, preferences, and decision-making ability [2,3,4].

According to the literature, the enforcement of involuntary psychiatric hospitalization varies widely among countries [1,5], ranging from 3.2% to 30% of all psychiatric hospitalizations [1]. In a study comparing the annual rates of involuntary hospitalization in Australia, New Zealand, and 22 European countries, the median rates vary from 14.5 per 100,000 people in Italy to 282 per 100,000 people in Austria [5].

This variation between countries might reflect socio-demographic and economic differences as well as different policies on mental health services and the progress of psychiatric reform. In some countries (e.g., Italy, Spain, United Kingdom), the health and mental health systems are more oriented towards community services, whereas in other countries (e.g., Poland), hospital health care dominates [6]. Despite these differences, there is a general increasing trend in involuntary admissions [1,7].

In Greece, there has been a continuous effort towards mental health reform in the direction of establishing a community-based mental health care system, including legislation changes, re-organization, and development of community mental health services. Despite these efforts, the deinstitutionalization process remains incomplete [8,9]. Hence, the rate of compulsory admissions in the country is still high. In recent years, involuntary hospitalization has shifted from a last resort to common practice [10]. According to recent studies in Greece, admissions to psychiatric hospitals without the patient’s consent range from 25% to 60% of psychiatric hospitalizations, while the prevalence of involuntary hospitalizations in the population is unknown [10,11,12]. The significantly higher rates of involuntary hospitalizations in Greece compared to most European countries render this issue in need of further systematic investigation [10].

The moral, social, and ethical dilemmas related to involuntary admissions have been studied thoroughly [13,14]. Τhe factors that may increase the probability of a patient with a mental disorder being admitted involuntarily to a psychiatric department is also an important research question. These factors can be divided into two broad categories. The first category includes those that concern a country’s mental health system and legislation [2,6,15]. However, it seems that legislation alone, although important, does not determine clinical practice. Legal regulations, quality of mental health care systems, and general attitudes of society must be considered along with other factors when studying involuntary admissions [2]. The second category includes patient-related factors, such as gender, nationality, age, educational level, socio-economic, marital, and employment status, as well as diagnosis and severity of psychopathology [1,16,17,18].

The aim of this study is to investigate the demographic and clinical factors possibly associated with the emergency psychiatric assessment of patients without their consent and its outcome in Greece. The study was carried out in the psychiatric department of the University General Hospital of Alexandroupolis (UGHA) in northeastern Greece.

## 2. Materials and Methods

### 2.1. Setting

**Figure 1:**
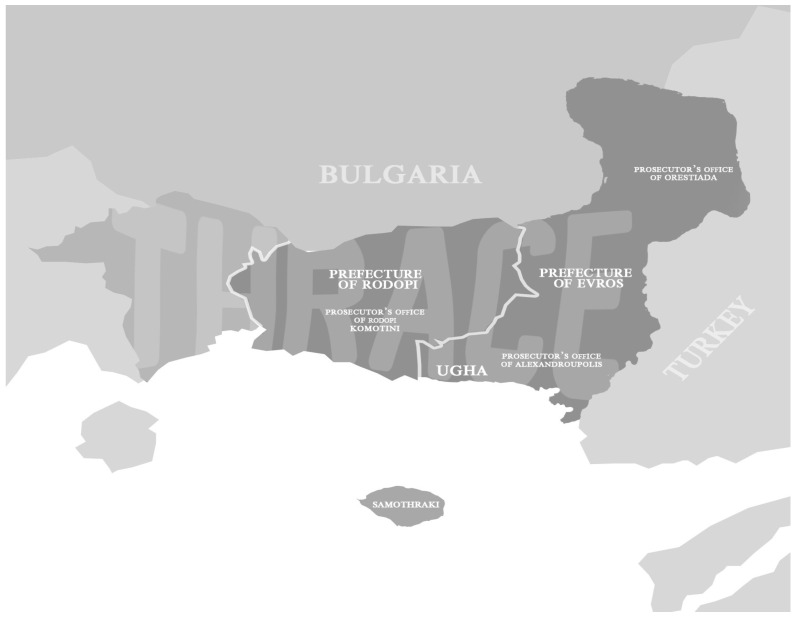
Mental Health Sector of Rodopi and Evros’ prefectures of Thrace, in northeastern Greece (time from Alexandroupolis to Orestiada and from Alexandroupolis to Komotini by car: 1 h 2 min and 43 min, respectively).

The psychiatric department of the University General Hospital of Alexandroupolis (UGHA) has 22 beds, which are usually fully occupied; sometimes there is even a 120% over-occupancy. It treats patients who belong to the Mental Health Sector of Rodopi and Evros’ prefectures of Thrace, in northeastern Greece (Figure 1). The orders for involuntary psychiatric assessments are issued by three prosecutorial authorities: i. Alexandroupolis (90,759 inhabitants in the prefecture of Evros), ii. Orestiada (57,188 inhabitants in the prefecture of Evros), iii. Rodopi in the city of Komotini (112,039 inhabitants in the prefecture of Rodopi). Μost psychiatric services are concentrated around the Alexandroupolis area. The remaining areas are covered by outpatient psychiatric clinics (only for the prefecture of Rodopi), a mental health center (only for the region of Orestiada), and mobile mental health units that visit several areas from once a week to once a month. About 40% of the population resides in rural areas.

According to Greek legislation, involuntary psychiatric assessments are demanded by the prosecutor following a request by close relatives, whoever has custody of the person, and/or the prosecutor themself. The assessment can result in 3 outcomes: i. admission (involuntary or voluntary), ii. immediate release when the hospitalization criteria are not met, and iii. temporary hospitalization for 48 h clinical observation and decision within this time period, after which the examinee will be discharged or will be treated involuntarily.

### 2.2. Sample

The sample consists of 191 individuals who were involuntarily brought in by police officers for psychiatric assessment in the Psychiatric Emergency Room (PER) of UGHA, during a one year period (1 March 2018 to 28 February 2019). It included 228 emergency psychiatric assessments in total (as 37 individuals were involuntarily re-examined). The sample was categorized by the Prosecutor’s Office that issued the order.

### 2.3. Procedure

This study is part of the “Study of Involuntary Hospitalizations in Greece (MANE)”, a multicenter research program that examined the process of and risk factors for involuntary admissions in Greece. The present study was carried out by using data extracted exclusively from hospital administrative records, with the agreement of the hospital. The following demographic and clinical data were extracted: gender, age, marital status, Prosecutor’s Office that ordered the assessment, previous visit to a psychiatric service, previous psychiatric follow-up, ICD-10 diagnosis, outcome of assessment (admission/release), type of hospitalization (voluntary/involuntary), and involuntary readmissions within a year. The ICD-10 diagnosis used for this analysis was collected from the examinee’s administrative record, specifically from the diagnosis reported in the official response letter from the hospital to the Prosecutor’s Office that had requested the involuntary assessment. All data were anonymized.

### 2.4. Statistical Analysis

Descriptive analysis was performed using frequencies, percentages, means, and standard deviations. T-tests and one-way ANOVA were used in order to allow for comparisons of continuous variables between subgroups, while χ^2^ tests and Fisher’s exact test for small numbers were used for comparisons between categorical variables. Univariate and multivariate logistic regression models were also run to assess associations between (a) outcome of examination (admission vs. release) and type of admission (voluntary vs. involuntary) as outcome variables and (b) various demographic and clinical characteristics.

Results were considered significant for *p* < 0.05. Statistical analysis was conducted using Statistical Package for Social Sciences (SPSS), version 24.0.

## 3. Results

### 3.1. Demographics

The sample consisted of 191 individuals. Seventy-four (39%) of them were referred to the hospital for involuntary assessment by the Alexandroupolis Prosecutor’s Office, 47 (25%) by the Orestiada Prosecutor’s Office, and 70 (37%) by the Rodopi Prosecutor’s Office (Table 1).

The majority of the sample were men (114 or 60%) and the mean age was 49 ± 16 years. Men appeared to be younger (47 ± 16 years) compared to women (52 ± 16) and the difference was at the borderline of statistical significance (*t*-test = 2.4, *p*-value = 0.046) (Table 1). More than half of the sample (96 or 53%) were single. There were statistically significant differences in marital status between men and women: more men than women were single (65% vs. 35% respectively), while 25% of women were married and 28% divorced or separated compared to 15% and 15% of men, respectively (χ^2^ test = 15.6, *p*-value = 0.001) (Table 1).

### 3.2. Assessments

Of the 191 individuals involuntarily examined at the PER of UGHA, 160 (84%) were assessed only once during the reference period. For the remaining 31 (16%), there was more than one involuntary assessment (28 were assessed twice, 1 three times, 1 four times, and 1 five times). This brought the total number of involuntary psychiatric assessments of our sample to 228.

Forty-two percent (42%) of examinees were diagnosed with “F20–29 schizophrenia, schizotypal and delusional disorders” (Table 2). In addition, a diagnosis of “F10–19 mental disorders and behavioral disorders due to the use of psychoactive substances” was assigned to 12% of the persons who were involuntarily assessed. For 25 individuals (13%), no specific diagnosis was given during their involuntary assessment. The frequency of patients with an “F20–29 schizophrenia, schizotypal and delusional disorders” diagnosis who were assessed in the PER more than once (11 or 18%) presented no significant difference compared to those with other diagnoses (20 or 18%, χ^2^ test = 0.73, *p*-value = 0.39).

Involuntary assessments were more frequent in the summer months of June (13% of all assessments), July and August (11% each), as well as in January (11%).

### 3.3. Outcomes following Involuntary Assessment

The most common outcome following involuntary assessment was hospital admission (133 or 70% of all individuals and 161 or 71% of all assessments), followed by immediate release from the PER of the hospital (43 or 23% of all individuals and 51 or 22% of all assessments). Fifteen patients (8%) (16 or 7% of all assessments) were discharged after having stayed in the hospital for 48 h. These brought the total number of examinees who were released to 58 (30% of individuals) or 67 releases (29% of all assessments) (Table 3).

Hospital admissions were mainly involuntary: 99 (74%) of admitted individuals or 117 (73%) of assessments resulted in hospitalization. Thus, in total, 51% of all involuntary assessments resulted in involuntary admissions.

### 3.4. Associations between Outcomes and Demographic and Clinical Characteristics

There were no statistically significant differences in the outcome of involuntary emergency assessment (admission/release) in relation to gender, age, or marital status. However, the frequency of admission was statistically significantly higher in patients referred from the Prosecutor’s Office of Orestiada (83%) than from the Prosecutor’s Offices of Alexandroupolis and Rodopi (69% and 61%, respectively, χ^2^ test = 6.21, *p*-value = 0.045).

There were statistically significant differences between diagnosis and outcome of involuntary emergency assessment (admission or release) (Fisher’s exact test *p*-value < 0.001). Almost all patients with a diagnosis of “F20–29 schizophrenia, schizotypal and delusional disorders” were subsequently admitted to the hospital (77 of 81, 66 of them involuntarily). On the other hand, most people referred for behaviors unrelated to a psychiatric condition were immediately released or discharged (23 of 25) (Table 2).

There were only 22 (17%) individuals among those admitted to the hospital who had no previous visit to a psychiatric service, compared to 111 (84%) who had visited a psychiatric service before the assessment, and the difference was statistically significant (χ^2^ test = 25.77, *p*-value < 0.001). In addition, 79 (59%) admitted participants had had a previous psychiatric follow-up (systematic or sporadic) compared to 53 (40%) who had no psychiatric follow-up, at least for the previous year, and the difference was again statistically significant (χ^2^ test = 18.2, *p*-value < 0.001). Moreover, among those released, 37 (67%) had never been followed up previously and 16 (29%) had been followed up either sporadically or systematically.

We subsequently ran logistic regressions, first with admission to or release from the hospital and then with voluntary or involuntary admission as outcomes (Table 4). Logistic regression models showed no statistically significant association between the outcome variables and any demographic characteristics. In terms of the Prosecutor’s Office, those coming from Orestiada’s had almost three times the odds of being admitted compared to those coming from Alexandroupoli’s, but the association was not statistically significant. The Prosecutor’s Office was not significantly associated with voluntary or involuntary admission. Those with “F20–29 schizophrenia, schizotypal and delusional disorders” diagnosis were almost nineteen times more likely to be admitted to the hospital compared to those with other diagnoses (OR = 18.56, 95% CI {6.35, 54.25}). The odds of involuntary admissions compared to voluntary ones also increased for those with an “F20–29” diagnosis (OR = 2.27, 95% CI {1.02, 5.07}). In addition, the odds of being admitted to the hospital were reduced by 81% for those with no previous contact with psychiatric services compared to those who had visited a psychiatric service before (OR = 0.18, {0.09, 0.38}). The odds of being admitted to the hospital increased for those who had been followed up systematically compared to those who had never been followed up before (OR = 6.79, 95% CI = {2.47, 18.64}) as well as for those who had been followed up sporadically (2.04 {0.92, 4.54}), but the latter was not statistically significant. When comparing voluntary to involuntary admissions, the association was not statistically significant.

In multivariate logistic regression, both no previous contact with psychiatric services and an “F20–29” diagnosis continued to be statistically significant, with admission to the hospital as an outcome variable. Specifically, the risk of hospitalization increased for those with a diagnosis of psychosis and decreased for those who had no prior contact with psychiatric services.

## 4. Discussion

The aim of the present study was to identify the demographic and clinical factors that are associated with involuntary assessment, as well as its outcome (release or admission, voluntary or involuntary admission), in northeastern Greece.

It is worth noting that a little more than half (51%) of the involuntary assessments resulted in involuntary admissions to the psychiatric department of UGHA. In the same period, rates of involuntary assessments turning into involuntary admissions were 96.9% in Athens and 88.5% in Thessaloniki, the two major urban Greek centers [12]. Μetropolitan areas probably face more challenges in terms of continuity of care, sectorized organization of mental health services, and integrated care provided by community services. The social, demographic, and organizational factors affecting involuntary admissions in those areas are potentially more complex than in regional–rural areas, such as Evros and Rodopi, and definitely necessitate further investigation.

Regarding demographic factors, previous studies have shown that male gender, single marital status, unemployment, and coming from a minority ethnic and linguistic background increase the possibility of involuntary admission [16,17,18]. Although in our study we did not find any statistically significant differences regarding the frequency of admissions or releases related to any demographic factors, there is a tendency for young, single men to be referred to hospital involuntarily.

A statistically significant association was found between the Prosecutor’s Office, as a source of referral for involuntary assessment, and admission rates, with admission rates being significantly higher for referrals from the Prosecutor’s Office in Orestiada, which is the furthest away from the hospital. In fact, this factor also represents the participants’ region of residence. Patients who live in the region of Alexandroupolis have easy access to mental health services that are provided by UGHA. Continuity of care, community networking, follow-up consistency, and early intervention are some of the features that are facilitated by the proximity of services and are important for curbing the rate of psychiatric hospital admissions, specifically involuntary admissions. Conversely, people with mental health problems living in remote areas, such as Orestiada (see Figure 1), face more challenges with regard to the above. Consistent with this finding, a study that was carried out in Thessaloniki, Greece, showed that involuntary admission rates were higher for patients living in regions away from the city [12]. A study carried out in the Canton of Vaud, Switzerland, also found that there is a variation between districts in terms of involuntary admission rates due to disparities in the availability of psychiatric services [19]. Notably, in the neighboring prefecture of Rodopi, where, similarly to Orestiada, mental health services are limited compared to those of Alexandroupolis, the outcome of the involuntary assessment is not affected. This is probably related to the fact that regions in the jurisdiction of the Prosecutor’s Office of Orestiada are more distant from the psychiatric department of UGHA, the only service that provides inpatient care in the two prefectures, than most of the regions in the jurisdiction of the Prosecutor’s Office of Rodopi. Moreover, accessibility to UGHA is hampered by the low-quality road network in the Orestiada region. Admissions are a common and standard practice for the prevention and treatment of relapses in patients residing in remote regions since mobile mental health units visit them from once a week to once a month. For these patients and their families, an involuntary procedure is often the only option, as they may not have the physical, practical, and financial resources to attend the hospital. Ιn these cases, the police actually operate as a psychiatric emergency medical service, a unit service that does not exist in Greece yet. In any case, this finding merits further investigation.

Involuntary assessments were more frequent in the summer months as well as in January. According to the literature, there is a correlation between seasonality and admissions for serious mental illness. The findings suggest a strong indication of a positive spring/summer correlation due to high environmental temperature [20,21,22,23]. Furthermore, during the summer period, as well as in January, professionals’ leaves of absence are common. Patients’ needs for treatment and care may not be met to a satisfactory level as there are fewer professionals working in outpatient psychiatric services. Therefore, relatives and social services approach the Prosecutor’s Office in order to ensure an assessment or an admission.

In line with international literature, we found that diagnosis was associated with the outcome of involuntary emergency assessment [16,17,18]. In particular, almost half of the involuntary examinees were diagnosed with psychosis (F20–29) and they were significantly more likely to be admitted to the hospital involuntarily compared to those with other diagnoses. These results could be explained by poor insight and a lack of awareness of the need for treatment in patients with psychosis during an acute phase [18,24]. Psychotic disorders are among the most severe and enduring mental health conditions. According to Gravier and Eytan (2011), social intolerance of deviant behaviors, which are common in patients with psychosis, leads to an increase in restrictive and coercive practices [25], thus the importance of community awareness is highlighted. Social factors, such as family burden and lack of supportive networks, may play an important role, often leading relatives and social services to approach the local Prosecutor’s Office [10]. In contrast to recent studies, organic mental disorders, intellectual disabilities, and comorbidity of addiction and psychosis were not found to be statistically associated with involuntary admission [17,18].

Regarding previous contact with mental health services, we found that the majority of those admitted to the psychiatric department had visited a mental health service prior to their involuntary assessment and more than half of them had previous psychiatric follow-up (systematic or sporadic). These findings could be related to the fact that mental health services in the region mainly follow up with people with severe mental disorders, such as psychosis. As mentioned above, these patients are at higher risk of being admitted involuntarily. This is consistent with the fact that patients with psychotic disorders have an increased risk of relapse, which can lead to psychiatric hospitalizations [26]. Even though professionals working in outpatient mental health care units in the two prefectures are in direct contact with the inpatient psychiatric department of UGHA and commonly refer patients with signs of relapse, attempting to support voluntary referral and hospitalization, it is notable that patients’ attendance at hospital mainly occurs involuntarily. This is probably related to the fact that the psychiatric department of UGHA is usually overcrowded, so planned voluntary admissions are delayed until a bed is available. These delays can lead to worsening of patients’ mental state or increasing the burden of their familial environment, rendering involuntary referral for assessment unavoidable. In line with the aforementioned, a study carried out in England showed that a reduction in psychiatric beds is associated with an increase in involuntary admissions [27].

Conversely, we found that 65% of examinees who were released or discharged from UGHA had no previous contact with psychiatric services. The absence of contact with mental health services was found to be an important factor in protecting from involuntary admission. Given that patients who are already followed up are those with the most severe and long-term psychopathology, as argued above, we may assume that those who are not being followed up are either new cases or cases of minor clinical significance who do not need hospitalization. This may be the reason for their release after involuntary assessment. This may also happen because professionals tend to recommend outpatient follow-up for people without severe symptoms in order to avoid stigma and possible negative outcomes associated with psychiatric hospitalization [28]. Subsequently, research on whether released examinees had any follow-up after their involuntary psychiatric assessment would be of interest.

The present study is one of the few studies in Greece regarding the outcome of involuntary attendance at a psychiatric department. However, it was not without limitations; the severity of symptoms was not assessed, and the diagnosis recorded was the one given during the psychiatric assessment in the PER and not the diagnosis given at the end of the admission, at discharge. The sample size is also relatively small.

## 5. Conclusions

Raising community awareness, as well as strengthening mental health services in remote areas, is of great importance. These services need to respond to the real psychiatric needs of the population and effective cooperation and networking with local social services should be promoted. The above, combined with the implementation of law in terms of creating a specialized service for the transport of patients to PER for psychiatric assessment when necessary, could reduce the tendency of the patients’ environment to approach the Prosecutor’s Office and the police when they feel that urgent treatment is needed for their loved one.

## Figures and Tables

**Table 1:** Demographic characteristics.

	Men (*n* = 114)	Women (*n* = 77)	Total (*n* = 191)	*p*-Value *
**Age**	47 ± 16	52 ± 16	49 ± 16	**0.046**
**Prosecutor’s Office**				0.998
Alexandroupolis	44 (39%)	30 (39%)	74 (39%)	
Orestiada	28 (25%)	19 (25%)	47 (25%)	
Rodopi	42 (37%)	28 (36%)	70 (37%)	
**Marital Status**				**0.001**
Married	16 (15%)	18 (25%)	34 (19%)	
Single	71 (65%)	25 (35%)	96 (53%)	
Divorced/Separated	16 (15%)	20 (28%)	36 (20%)	
Widow	7 (6%)	9 (13%)	16 (9%)	

* *t*-test.

**Table 2:** ICD-10 diagnosis by the outcome of the involuntary emergency psychiatric assessment.

Diagnosis (ICD-10)	Admitted *n* (%)	Released *n* (%)	Total *n* (%)
F00–09	12 (9%)	6 (10%)	18 (9%)
F10–19	14 (11%)	9 (16%)	23 (12%)
F20–29	77 (58%)	4 (7%)	81 (42%)
F30–39	15 (11%)	2 (3%)	17 (9%)
F40–49	4 (3%)	1 (2%)	5 (3%)
F60–69	8 (6%)	10 (17%)	18 (9%)
F70–79	1 (1%)	2 (3%)	3 (2%)
Χ60–84	0 (0%)	1 (2%)	1 (1%)
Other	2 (2%)	23 (40%)	25 (13%)
Total	133	58	191

**Table 3:** Outcomes following involuntary assessments.

	Examinees (*n* = 191)	% over Total Examinees	Assessments (*n* = 228)	% over Total Assessments
**Outcome**				
Admission				
Involuntary	99	52%	117	51%
Voluntary	34	18%	44	19%
Total Admissions	133	70%	161	71%
Release				
Immediate	43	23%	51	22%
After 48 h	15	8%	16	7%
Total releases	58	30%	67	29%

**Table 4:** Univariate and multivariate logistic regression with admission or release and voluntary or involuntary admission as outcome variables.

	Admission vs. Release	Voluntary vs. Involuntary
	OR {95% CI}	OR {95% CI}
**Univariate**		
Schizophrenia (yes vs. no)	**18.56 {6.35, 54.25}**	2.27 {1.02, 5.07}
First visit (yes vs. no)	**0.19 {0.09, 0.38}**	1.12, {0.38, 3.29}
Follow-up		
Never	{Ref. *}	{Ref. *}
Sporadically	2.04 {0.92,4.54}	1.24 {0.52, 2.98}
Systematically	6.79 {2.47, 18.64}	0.85 {0.38, 1.92}
Prosecutor’s Office		
Alexandroupolis	{Ref. *}	{Ref. *}
Orestiada	2.20 {0.89, 5.44}	2.33 {0.75, 7.20}
Rodopi	0.72 {0.36, 1.43}	0.62 {0.25, 1.50}
**Multivariate**		
Schizophrenia (yes vs. no)	**13.85 {4.63, 41.44}**	**2.37 {1.05, 5.33}**
First visit (yes vs. no)	**0.25 {0.11, 0.55}**	1.79 {0.54, 5.91}

* Reference group.

## Data Availability

Data are contained within the article.
